# Integrated generation of vortices and frequency conversion with metasurfaces

**DOI:** 10.1038/s41377-025-01831-z

**Published:** 2025-04-02

**Authors:** Jinyong Ma, Kenneth B. Crozier, Andrey A. Sukhorukov

**Affiliations:** 1 ARC Centre of Excellence for Transformative Meta-Optical Systems, Department of Electronic Materials Engineering, Research School of Physics, The Australian National University, Canberra, 2600 ACT Australia; 2 ARC Centre of Excellence for Transformative Meta-Optical Systems, School of Physics, University of Melbourne, Melbourne, 3010 VIC Australia; 3 ARC Centre of Excellence for Transformative Meta-Optical Systems, Department of Electrical and Electronic Engineering, University of Melbourne, Melbourne, 3010 VIC Australia

**Keywords:** Nanophotonics and plasmonics, Nonlinear optics

## Abstract

The generation of optical vortices in compact systems and across different spectral regions can open new horizons for their applications in end-user devices. Latest advances in the design and fabrication of optical metasurfaces made of a quadratically nonlinear material enable highly precise creation of vortices with different topological charges at the second-harmonic frequency, with the potential to obtain various other structured states of light.

Optical vortices have attracted continued research interest for more than 35 years^1^ due to their intriguing features and practical applications. The vortices can carry orbital angular momentum (OAM), while at their core the intensity drops to zero due to the presence of a phase singularity, as shown in Fig. 1a. Such structured states of light can facilitate the trapping and rotation of particles, enhance imaging functionality, and increase the capacity of communication links^2,3^. There is currently a trend toward the miniaturisation of optical systems, driven largely by new applications enabled by their incorporation into platforms such as drones and handheld consumer electronics. This motivates the development of ultra-compact and lightweight optics for the preparation and manipulation of structured light including vortices.

**Fig. 1:**
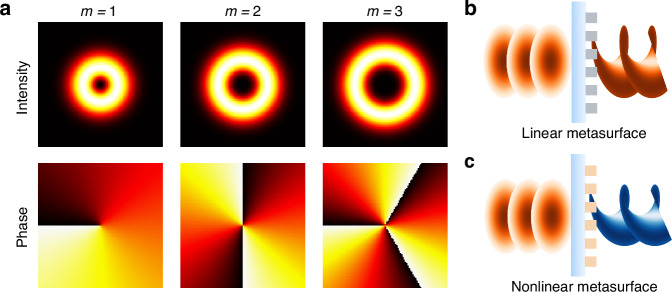
**a** Intensity and phase profile of optical vortices at different topological charges *m*. **b** Generation of optical vortices with a linear metasurface. **c** Generation of second and higher harmonic optical vortices with a nonlinear metasurface

Metasurfaces incorporating ultrathin nanostructures have enabled unprecedented control over the polarization and phase of light at the nanoscale [Fig. 1b], with a practical potential to replace bulky optics for generating optical vortices on demand^4^. The first work arranged plasmonic meta-atoms with spatially varying orientations, thereby imprinting helicoidal equal-phase wavefront onto the incident light^5^. Later on, the J-plate metasurface composed of rectangular units with varying sizes and orientations achieved the conversion of any chosen spin angular momentum to a target OAM, overcoming the limitation of the geometric phase^6^. Complex-amplitude metasurfaces facilitated holography with multiplexing of up to 200 independent OAM channels^7^. Recent advances in cascaded metasurfaces push this direction further, achieving high-efficiency and high-purity OAM multiplexing with record high Laguerre polynomial orders^8^.

The light wavefront can also be nontrivially transformed in the process of nonlinear frequency conversion. It is well established that OAM can be doubled through second-harmonic generation in homogeneous nonlinear crystals^9^. Furthermore, spatially modulated nonlinear photonic crystals enable the generation of arbitrary second-harmonic OAM even from input beams without angular momentum^10^. Nonlinear optical metasurfaces^11^, containing specially optimized nanopatterns that can manipulate both phase and amplitude in the process of frequency conversion, allow even more flexibility in OAM manipulation combined with second^12–14^ or third harmonic generation^15,16^, as sketched in Fig. 1c.

A recent paper in *Light: Science & Applications*, by Laure Coudrat and a team led by Aloyse Degiron and Giuseppe Leo from the Paris Cité University and CNRS, France^17^, reports a significant advance in the generation of vortices from dielectric metasurfaces made of a quadratically nonlinear material AlGaAs, which were designed such that the second-harmonic phase was precisely controlled in space to create the desired output wavefront. A set of metasurfaces was fabricated and used in experiments to generate second-harmonic vortices with topological charges between 1 and 10 from an unstructured input pump beam. Importantly, the purity of vortices was found to be very high. Additionally, the nonlinear frequency conversion was resonantly enhanced by 50 times compared to an unstructured AlGaAs membrane, while the absence of optical losses in the nonlinear material is a significant benefit compared to the previously explored plasmonic structures.

The high precision with which the nonlinear vortex beam was generated was achieved by making the AlGaAs nanoresonators in the form of half-elliptic cylinders^17^ as shown in Fig. 2a. This is an innovative approach that is a nontrivial evolution of the nanochair resonators previously explored by the same research group and collaborators^18^, allowing for dramatic simplification of the fabrication process.

**Fig. 2:**
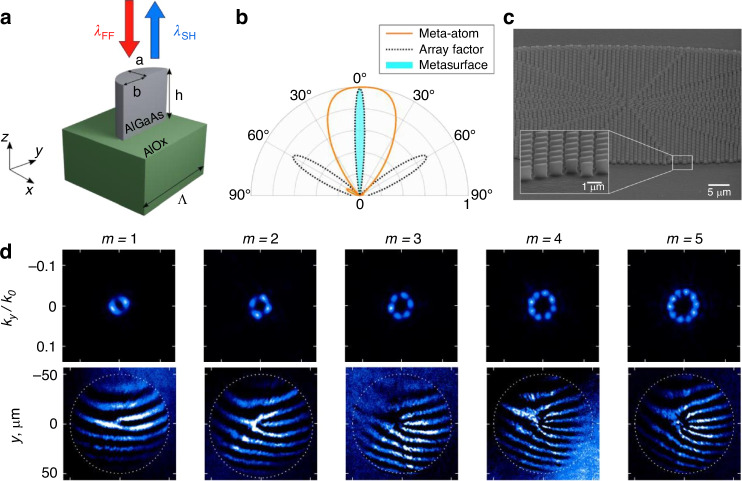
**a** Sketch of the nonlinear nanoresonator. **b** Characteristic second-harmonic radiation diagram in the *xz*-plane of a single meta-atom shown in figure **a** (orange), array factor for a sample metasurface with 8 identical nanoresonators in the *x*-direction (dashed black), and product of the two estimating the emission from a metasurface (light blue). **c** SEM image of the metasurface designed for the generation of second-harmonic vortex with charge 10. **d** Measured intensities (top) and interference patterns (bottom) of on-demand vortex generation from the metasurface, where *m* is the topological charge. The figures are reproduced from ref. ^17^

Remarkably, this geometrically simple design offers an elegant solution to the problem of controlling the directionality of second-harmonic, facilitating its efficient emission in the forward direction [Fig. 2b]. Simultaneously, the second-harmonic phase can be controlled over the full 2*π* range by continuously tuning the dimensions of the elliptical axes (*a* and *b* in Fig. 2a) and using two orientations of the nanoresonators at 0° or 180°. Then, the nanoresonator patterns were combined in a metasurface according to the desired second-harmonic output phase profile [Fig. 2c].

The experimental results demonstrated the generation of second-harmonic vortices with various topological charges from the input pulses of 160 fs duration at 1550 nm, using the specially designed metasurfaces^17^. The measured output intensities for the charges in the range 1 to 5 are reproduced in the top row of Fig. 2d, featuring a characteristic ring profile as expected theoretically. The interference measurements shown in the bottom row of Fig. 2d reveal fork structures with the number of maxima around a dislocation consistent with the different vortex charges. The intensity profiles have visible modulations around the ring, which appear due to the presence of oppositely charged vortices. This happened due to the small variations of the second-harmonic generation efficiency between the resonators of different shapes and orientations comprising the metasurface. Nevertheless, the experimentally measured vortex purity reached 80%, while the undesired oppositely charged vortex contribution was only 2% for a vortex with charge 10. It is anticipated that this performance can be further improved by optimizing the metasurface design and nanofabrication procedures.

The latest results suggest new opportunities with nonlinear metasurfaces for highly precise and flexible wavefront control at optical harmonics^15,19**–**21^ by using the nanoresonators with variable dimensions and orientations following ref. ^17^. While the pump beam and second-harmonic vortices in the latter study had a linear polarization, involving the polarization degree together with an optimized nonlinear response can allow for tunable vortex generation^22^. Another active research direction is the generation of photons with quantum entanglement in OAM^23,24^, which can be potentially tailored by structuring a nonlinear metasurface.

In most previous works, metasurfaces for vortex generation were designed to take a conventional Gaussian laser beam as the input. On the other hand, there is exciting progress in the development of lasers that directly generate structured light, whose spatial profiles of amplitude, phase, and polarization can be tailored to suit particular applications. One of the pioneering works on this topic was by Okaya^25^ in 1964, just a few years after the invention of the laser. Okaya showed that single or crossed tungsten wires inserted into the laser cavity allowed mode selection, enabling the user to choose the lasing mode to be TEM_1,2_, TEM_2,2_, etc. Since then, the field of lasers that generate structured light has burgeoned, see a recent review^26^. A notable example is by Liu et al.^27^, who experimentally demonstrated ultrashort (*<*650 fs) and high power (up to several hundred milliwatts) vortex pulses with the topological charge up to 30 from a low cost and compact laser source. Their Yb:KGW laser generated high order Hermite-Gaussian (HG) modes via careful control over the pumping scheme (off-axis and angle-based non-collinear). The HG modes were converted to vortices (Laguerre-Gaussian modes) by an astigmatic mode converter outside the laser cavity. These advances suggest new prospects in combination with metasurfaces, which can flexibly transform between different spatial modes, and also perform frequency conversion of femtosecond pulses to access broader spectral regions.
